# Molecular etiology study of hearing loss in 13 Chinese Han families

**DOI:** 10.3389/fneur.2022.1048218

**Published:** 2022-11-23

**Authors:** Lianhua Sun, Zhengyu Lin, Xiaowen Wang, Jiali Shen, Yue Li, Yuyu Huang, Jun Yang

**Affiliations:** ^1^Department of Otorhinolaryngology-Head and Neck Surgery, Xinhua Hospital, Shanghai Jiaotong University School of Medicine, Shanghai, China; ^2^Shanghai Jiaotong University School of Medicine Ear Institute, Shanghai, China; ^3^Shanghai Key Laboratory of Translational Medicine on Ear and Nose Diseases, Shanghai, China

**Keywords:** deafness, targeted sequencing, whole-exome sequencing, gene mutation, etiological analysis

## Abstract

Hearing loss affecting about 2/1000 newborns is the most common congenital disease. Genetic defects caused approximately 70% of patients who have non-syndromic hearing loss. We recruited 13 Chinese Han deafness families who tested negative for *GJB2, SLC26A4*, and mitochondrial 12S rRNA. The probands of each family were performed whole-exome sequencing (WES) or targeted next-generation sequencing (NGS) for known deafness genes to study for pathogenic causes. We found four novel mutations of *CDH23*, one novel mutation of *MYO15A*, one novel mutation of *TMC1*, one novel mutation of *PAX3*, and one novel mutation of *ADGRV1*, one novel CNV of *ADGRV1*, and one novel CNV of *STRC*. Hearing loss is a highly hereditary and heterogeneous disease. The results in the limited samples of this study show that Usher and Waardenburg syndrome-related genes account for a major proportion are strongly associated with Chinese Han hearing loss patients negative for *GJB2, SLC26A4*, and mitochondrial 12S rRNA, followed by *STRC* resulting in mild to moderate deafness.

## Introduction

Hearing loss is the most common congenital disease affecting about 2/1,000 newborns (1). Approximately 70% of non-syndromic hearing loss is caused by genetic defects (2). Autosomal recessive non-syndromic deafness, the most common form of hearing loss, is usually pre-lingual and accounts for 80% of non-syndromic hereditary deafness. Autosomal dominant non-syndromic deafness, which is often post-lingual, accounts for the remaining 20% (3). Mitochondria and X-linked inheritance account for only 1–2% of non-syndromic deafness. About 30% of genetic deafness is associated with about 700 symptoms described to date, leading to syndromic deafness (4). To date, more than 44 syndromic deafness genes and 100 non-syndromic deafness genes have been mapped (http://hereditaryhearingloss.org).

Currently, next-generation sequencing (NGS) is increasingly applied in clinics to enable accurate diagnosis. In this study, we recruited 13 Chinese Han deafness families negative for *GJB2, SLC26A4* and mitochondrial 12S rRNA. The probands of each family were performed targeted NGS for known hearing loss genes or whole-exome sequencing (WES) to study for pathogenic causes.

## Subjects and methods

### Subjects collection and audiological evaluations

Thirteen patients (HL1~13) were recruited from the Department of Otorhinolaryngology-Head and Neck Surgery of Xinhua Hospital affiliated with Shanghai Jiaotong University School of Medicine. Informed consent was approved from all subjects to participate in this study from October 1, 2018 to December 31, 2020. For child participants, written consent will be sought from their parents or guardians. All patients had a detailed medical history and a thorough examination to rule out noise, trauma, pregnancy infection, and other non-genetic factors. All affected subjects were evaluated by audiological examinations, including otoscopy, pure-tone audiometry (PTA), distortion product otoacoustic emissions (DPOAEs), and auditory brainstem response (ABR). Magnetic resonance imaging (MRI) was performed on the HL13 proband. The research was approved by the Ethics Committee of Xinhua Hospital affiliated to Shanghai Jiaotong University School of Medicine (No. XHEC-D-2021-060).

### Targeted NGS

Genomic DNA of all family members was extracted from whole peripheral blood leukocytes. Using polymerase chain reaction (PCR) amplification, *GJB2, SLC26A4*, and the mitochondrial 12S rRNA exon were directly sequenced first in 13 probands. A panel of 415 hearing loss–related genes was performed by targeted NGS in 12 probands excluding HL6 (Supplementary Table 1). Data processing including targeted gene capturing, filtering of multiple databases for variations, and bioinformation analysis was previously reported in detail (5). Potential causative mutations, which were detected by targeted NGS, were identified for each proband using Sanger sequencing. Where possible, a co-segregation analysis of all family members was also conducted.

### Whole exome sequencing

The whole exome sequence of the HL6 proband was sequenced in the Illumina platform by the NextSeq500 sequencer, and the obtained reads by whole exome sequence were mapped to the human genome reference sequence hg19.

### SNP arrays

We used SNP arrays to detect the chromosomal regions of CNV identified by targeted NGS in the HL5. SNP arrays were performed as previously reported in detail (5).

### Multiplex ligation-dependent probe amplification

The SALSA^®^ MLPA^®^ probe mixes P461-A1 DIS (MRC-Holland, Amsterdam, The Netherlands) was used to identify deletion/duplication of *STRC-CATSPER2* in HL6 and HL7 family members, according to the manufacturer's instructions. The PCR amplification products were analyzed on ABI 3500 Genetic Analyzer (Life-Technologies, Carlsbad, CA) using Gene Marker 1.91 software (Soft Genetics, State College, PA).

## Results

### Clinical manifestations

There are eight female and five male probands. Those affected individuals ranged in age from 14 months to 49 years. Patients from 13 Chinese families all had congenital, bilateral, and sensorineural hearing loss. They all come to the doctor because they have failed newborn hearing screening or were diagnosed with hearing abnormalities in infancy. Their hearing loss was relatively stable, with the exception of a mild progression of HL5 proband. Hearing loss was defined as varying degrees, including moderate, severe, and profound hearing loss. Click on the auditory brainstem response thresholds was 60–70 dBs for HL5, 40 dBs for HL6, 60–70 dBs for HL7, 40 dBs for the left ear, and 90 dBs for the right ear of HL11 proband. The other probands had profound hearing loss. The HL5 proband was diagnosed with retinitis pigmentosa by an ophthalmologist as a teenager including symptoms of small vision, night blindness, and amblyopia. The HL10 proband had a heterochromia iridis, and her father had a heterochromia iridis but no hearing loss. The HL11 proband had excessive freckles, and his father had excessive freckles but no hearing loss. The HL12 proband had hydronephrosis. Ear malformation was observed in the HL13 proband by MRI, including abnormal enlargement of inner ear canal, bone defect of cochlear apex and skull base, and cerebrospinal fluid.

### Genetic findings

**Figure 1:**
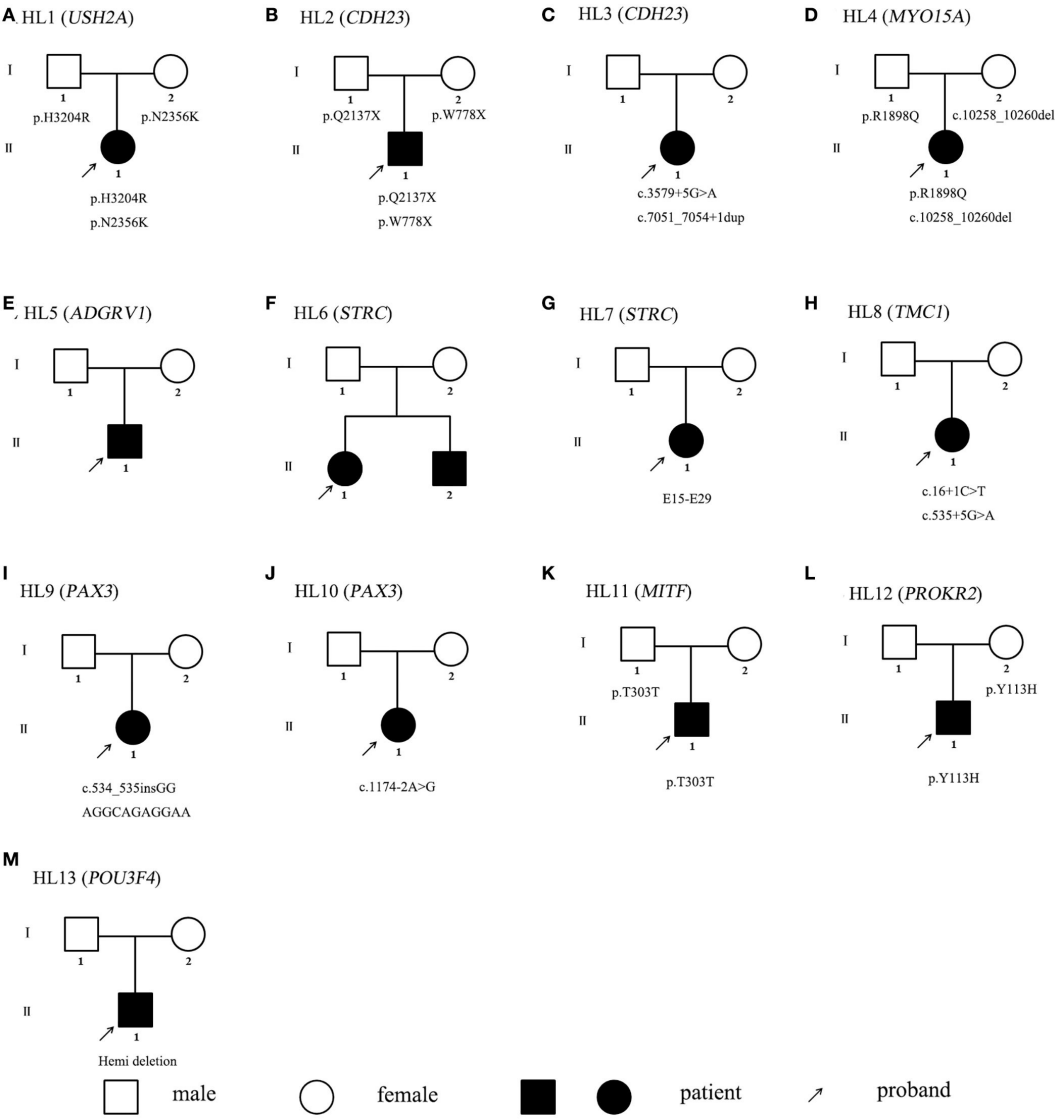
**(A–M)** Pedigrees of HL1-13.

**Figure 2:**
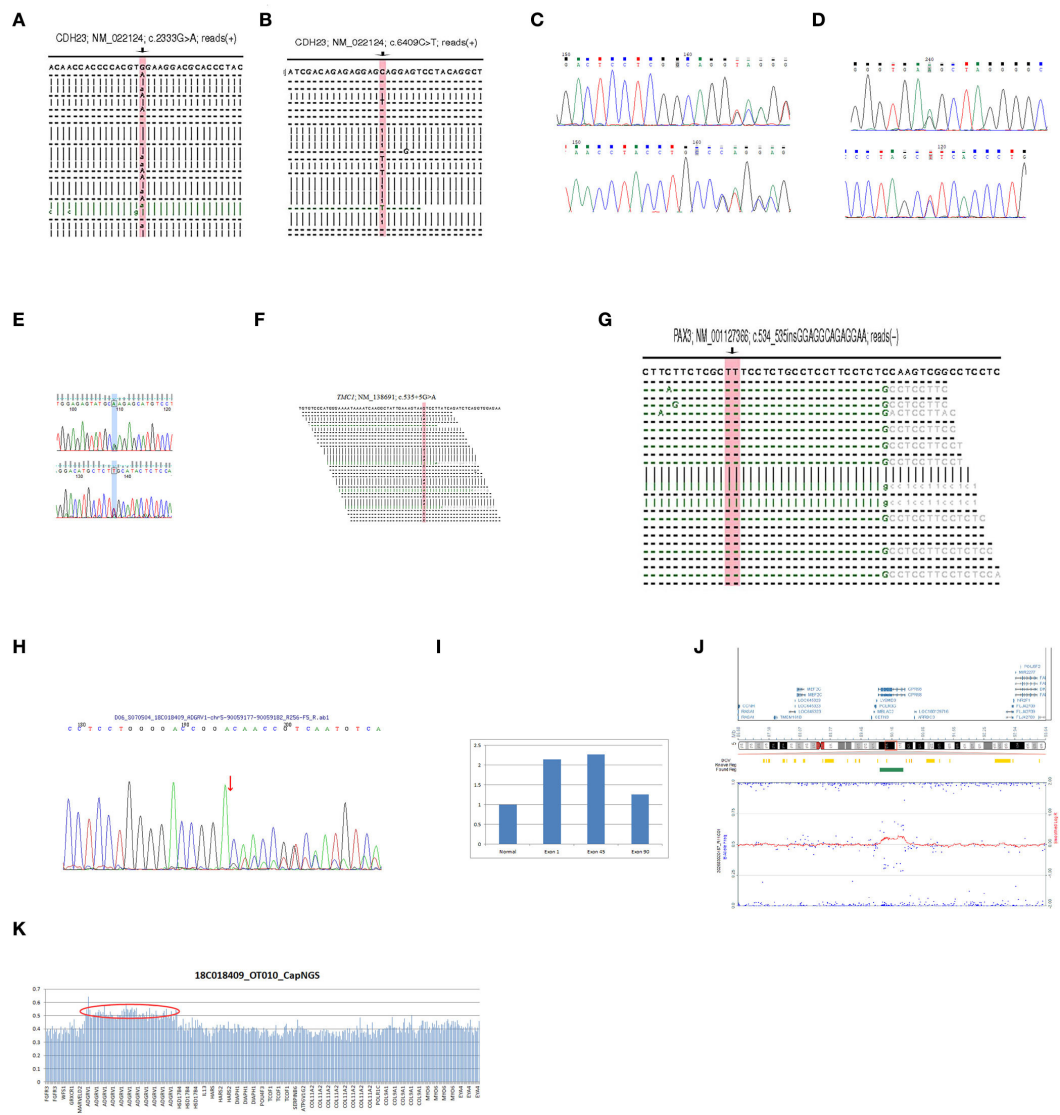
**(A)** The NGS result of the mutation c.2333G > A in *CDH23* of HL2 proband. **(B)** The NGS result of the mutation c.6409C > T in *CDH23* of HL2 proband. **(C)** The Sanger sequencing result of the mutation c.3579 + 5G > A in *CDH23* of HL3 proband. **(D)** The Sanger sequencing result of the mutation c.7051_7054 + 1dup in *CDH23* of HL3 proband. **(E)** The Sanger sequencing result of the mutation c.5693G > A in *MYO15A* of HL4 proband. **(F)** The NGS result of the mutation c.535 + 5G > A in *TMC1* of HL8 proband. **(G)** The NGS result of the mutation c.534_535insGGAGGCAGAGGAA in *PAX3* of HL9 proband. **(H)** The Sanger sequencing result of the mutation c.12177_12181delGGTTG in *ADGRV1* of HL5 proband. **(I)** The qPCR results of the exon 1, 45, and 90 in *ADGRV1* of the HL5 proband contrast to normal. **(J)** The SNP array result of the proband HL5. **(K)** The NGS result of the HL5 proband.

To detect possible causative variations by target NGS or WES, nonsynonymous variants were filtered, with minor allele frequencies greater than 0.005 for autosomal recessive, and minor allele frequencies greater than 0.0005 for autosomal dominant. Candidate causative mutations are summarized in Table 1. In 8 recessive families, bi-allelic mutations were found in known deafness genes, part of which were identified by parental genotyping including p.H3204R and p.N2356K in *USH2A* (OMIM 608400), p.Q2137X and p.W778X in *CDH23* (OMIM 605516), c.3795 + 5 G>A and c.7051_7054 + 1dup in *CDH23* (OMIM 605516), p.R1898Q and c.10258_10260 del in *MYO15A* (OMIM 602666), c.12177_12181delGGTTG and a duplication in *ADGRV1* (OMIM 602851), partial or whole gene deletion in *STRC* (OMIM 606440), c.16 + 1C>T and c.535 + 5G>A in *TMC1* (OMIM 606706) (Table 1). In 4 dominant families, four heterozygous variants associated with dominant deafness were identified, including c.534_535 in GGAGGCAGAGGAA in *PAX3* (OMIM 606597), c.1174-2A > T in *PAX3* (OMIM 606597), p.T303T in *MITF* (OMIM 156845), and p.Y113H in *PROKR2* (OMIM 607123), as well as partial co-segregating with the phenotype (Figure 1). In the HL13 proband, we detected the hemizygous deletion of *POU3F4* gene by targeted sequencing, which is consistent with the clinical phenotype of the patient. The co-separation of the reported mutations was confirmed from the hearing phenotype of the extended family members by Sanger sequencing (Figures 1, 2). Of the 19 mutations identified in this study, 10 were reported to be associated with deafness for the first time (Table 1, Figure 2).

**Table 1:** The gene mutation of HL1-13.

**Gene**	**Mutation type**	**Nucleotide change (transcript version)**	**Amino acid change**	**InterAcmg**	**Mutationtaster**	**Pathogenic grade**	**SIFT (score)**	**Allele frequency in controls**	**References**
Autosomal recessive								
USH2A	Missense	c.9611A>G (NM_206933)	p.H3204R	PM3_Strong, BP4	Polymorphism (1)	Uncertain	Tolerated (0.052)	0/1000	25649381
	Missense	c.7068T>G (NM_206933)	p.N2356K	PM1, BP4	Disease_causing (0.937)	Uncertain	Damaging (0.005)	0/1000	30245029
CDH23	Stop coden	c.2333G>A (NM_022124)	p.W778*	PVS1, PM2	Disease_causing_automatic (1)	Likely_pathogenic	-	0/1000	Novel
	Stop coden	c.6409C>T (NM_022124)	p.Q2137*	PVS1, PM2	Disease_causing_automatic (1)	Likely_pathogenic	-	0/1000	Novel
CDH23	Splicing	c.3579+5G>A (NM_022124.5)	-	PM2	-	Uncertain	-	0/1000	Novel
	Splicing	c.7051_7054+1dup (NM_022124.5)	-	PVS1, PM2	-	Likely pathogenic	-	0/1000	Novel
MYO15A	Missense	c.5693G>A (NM_016239.3)	p.R1898Q	PM2, PP3	Disease_causing (1)	Uncertain	Damaging (0.011)	0/1000	Novel
	Frameshift	c.10258_10260del TTC(NM_016239.3)	p.F3420 fs*	PM3_Strong, PM2, PM4	-	Likely pathogenic	-	0/1000	31250571
ADGRV1	Frameshift	c.12177_12181delGGTTG (NM_032119)	p.V4060 fs*12	PVS1,PM2	-	Likely pathogenic	-	0/1000	Novel
	CNV (whole)	chr5: 89887683 - 90431463	-	-	-	-	-	-	Novel
STRC	CNV (whole)	chr15:43888567-43988112	-	-	-	-	-	-	Novel
STRC	CNV (E15-29)	chr15:43891839-43902647	-	-	-	-	-	-	26969326
TMC1	Splicing	c.16+1C>T (NM_138691)	-	PVS1,PM3_Strong,PM2	Disease_causing (1)	Pathogenic	-	0/1000	25525159
	Splicing	c.535+5G>A (NM_138691)	-	PM2	-	Uncertain	-	0/1000	Novel
Autosomal dominant								
PAX3	Frameshift	c.534_535insGGAGGCAGAGGAA (NM_001127366)	p.Q178 fs*29	PVS1,PM2	-	Likely pathogenic	-	0/1000	Novel
PAX3	Splicing	c.1174-2A>T (NM_181457)	-	PVS1, PM3_Strong, PM2	Disease_causing (1)	Pathogenic	-	0/1000	27759048
MITF		c.909G>A (NM_000248)	p.T303T	PM3_Strong,PM2	-	Likely pathogenic	-	0/1000	29986705
PROKR2	Missense	c.337T>C (NM_144773)	p.Y113H	BS1, BS2	Disease_causing (1)	Benign	Damaging (0)	0/1000	30576231
X-linked recessive								
POU3F4	whole deletion	-	-	-	-	-	-	-	27577114

-, No information; *, stop coden.

**Figure 3:**
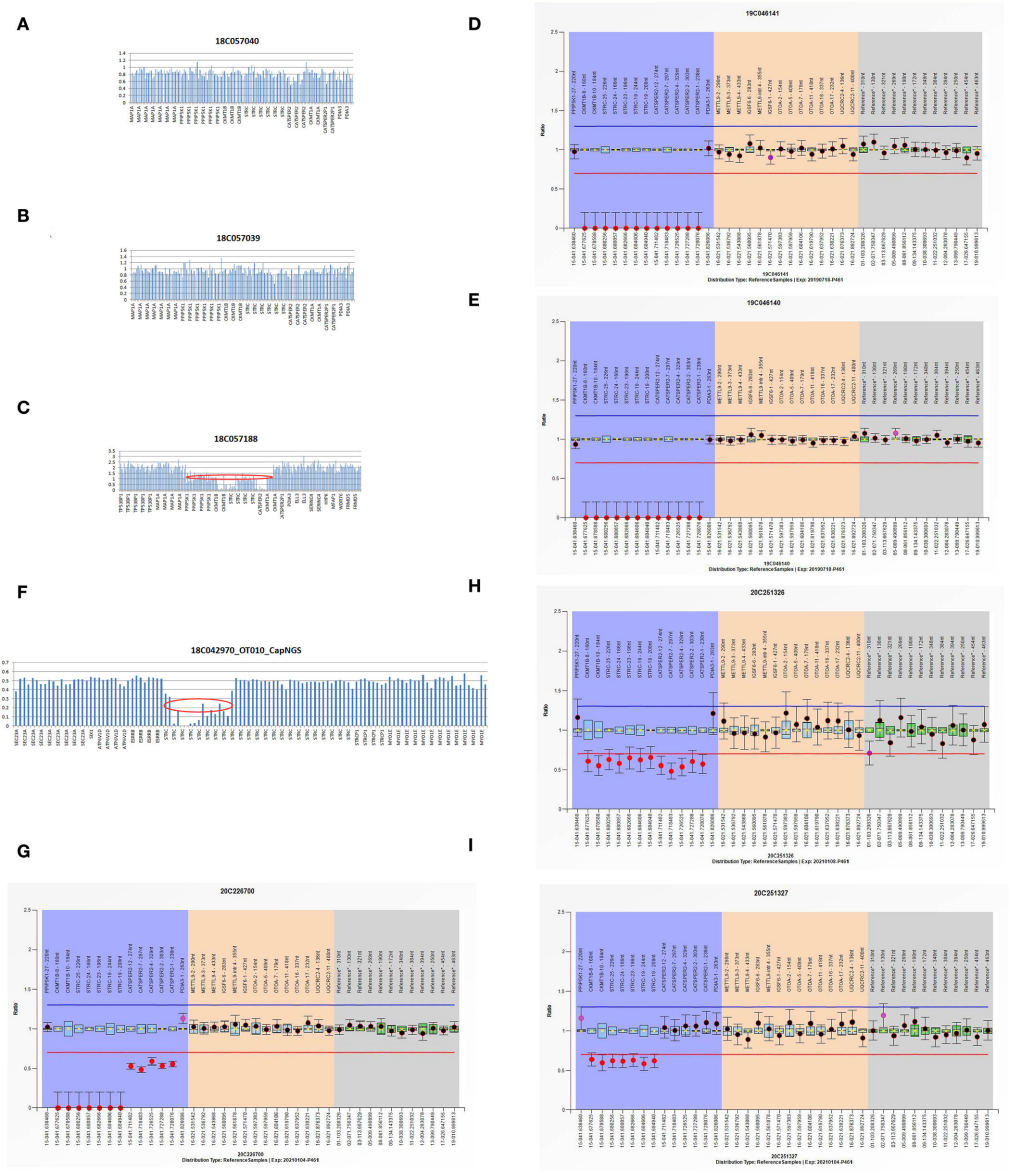
**(A–C**) The WES results of I:1, I:2, and II:1 in HL6 family. **(D,E)** The MLPA results of II:1 and II:2 in HL6 family. **(F)** The NGS result of HL7 proband. **(G–I**) The MLPA results of II:1, I:2, and I:1 in HL7 family.

Using targeted NGS and SNP arrays, the HL5 proband was found to replicate approximately 544 kb in chromosome region 5q14.3 [arr5q14.3 (89887683-90431463) X3] (Figure 2). Whole exon sequencing revealed a 99 kb of copy number variation in the HL6 proband at 15q15.3 (43888567-43988112), including *STRC, CKMT1A, CKMT1B*, and *CATSPER2* genes. Using MLPA, we found that the sibling in the HL6 family with the same symptoms also had the same CNV (Figure 3). In the HL7 family, the results of WES and MLPA revealed that the proband had a homozygous deletion in exon 8 and 10 of *CKMT1B* gene, and exon 19, 23–25 of *STRC* gene, heterozygous deletion of exon 1, 2, 4, 7, and 12 of *CATSPER2* gene. Using MLPA, we detected heterozygous deletion of exon 8, 10 of *CKMT1B* gene, and exon 19, 23–25 of *STRC* gene in the father of the proband; heterozygous deletion in exon 8 and 10 of *CKMT1B* gene, exon 19, 23–25 of *STRC* gene, and exon 1, 2, 4, 7, and 12 of *CATSPER2* gene in the mother of the proband (Figure 3).

## Discussion

In *CDH23*, homozygous nonsense, frameshift, some missense and splice site mutations, or compound heterozygotes combined of these above USH1D alleles are considered hypomorphic alleles with no sufficient retinal, vestibular, and auditory cochlear function leading to USH1D. Conversely, missense mutations in *CDH23* are associated with non-syndromic DFNB12 deafness. The DFNB12 allele maintains normal retinal and vestibular function and is dominant to the USH1D allele phenotypically, even in the presence of the USH1D allele (6). In this study, patients were young at the time of diagnosis. While there are no obvious vestibular and retina symptoms, based on the above principles, we can make a USH1D diagnosis in this study due to the nonsense and splice site mutations of *CDH23* in the proband HL2 and HL3. *ADGRV1* is a pathogenic gene of USH2C, which is belonging to USH2. The primary clinical manifestation of USH2 is congenital moderate to severe deafness, and onset of retinitis pigmentosa within 1–20 years of life, but without vestibular impairment. Besnard et al. (7) concluded that *ADGRV1* mutations account for 6.4% and a small but significant proportion of mutations that cause USH2. Newly research suggests gene dysfunction associated with Usher syndrome is the second-leading genetic cause of hereditary sensorineural hearing loss after connexin dysfunction (8). In this study, because the probands of HL1, 2, and 3 were still young and only showed hearing problems, they will develop into Usher syndrome in the future. The proband HL5 was over 30 years old, and he showed typical symptoms of the Usher syndrome, including hearing loss, small vision, night blindness, and amblyopia.

Of all congenital sensorineural deafness, *STRC* mutations reported for the first time in 2001 are currently estimated to account for ~5–6% (9). However, given variable *STRC* allele frequencies existing in different races, there may be a higher proportion, and an increasing number of cases are reporting copy number variation relevant to clinical in the *STRC* locus (10). *STRC*, with 99.6% coding sequence identity, is closely linked to the pseudogene and is a challenge for the analysis. The gene *CATSPER2*, a neighboring gene to *STRC*, is responsible for sperm motility and leads to deafness infertility syndrome in males, most commonly with sequential deletion of both *STRC* and *CATSPER2* genes. Women with this serial loss only suffer from hearing loss (11). The study indicates that, after the *GJB2* gene which is the majority of mild to moderate inherited deafness, *STRC* deletion accounts for the second most common cause (12). Due to racial differences and insufficient attention, the role of *STRC* gene mutations in the pathogenic of hereditary deafness in China is less reported. The results show that copy number variation of *STRC* gene is not uncommon in clinics. Next-generation sequencing can identify such cases. Combining with the MLPA method, patients can be accurately diagnosed with the *STRC* gene mutation, which requires us to pay more attention to its pathogenicity in the clinic.

Waardenburg syndrome is susceptible to being misdiagnosed as autosomal recessive due to *PAX3* spontaneous mutation and ignores *MITF*-related freckle phenotype. It is a *de novo* mutation c.534_535ins GGAGGCAGAGGAA of *PAX3* in the HL9 proband. C.1174-2A > T in *PAX3* of the HL10 proband and c.909G >A in *MITF* of the HL11 proband were inherited from their fathers, respectively, with heterochromia iridis or excessive freckles but no hearing loss.

DFNX2 (X-linked deafness type 2), with clinical features, typically include progressive mixed hearing loss, stapes fixation, and temporal bone anomalies, is the most common type of X-linked deafness in humans (13, 14). The *POU3F4* mutations account for approximately 50% of genetic causes of DFNX2 (15). Affected men showed mixed hearing loss or less commonly, only sensorineural hearing loss. Typical manifestations of MRI are characterized by hypoplasia of the cochlear base, thickening of the base of stapes floor, loss of the bony modiolus, expansion of internal acoustic meatus, and abnormally wide communication between the cochlear base and the auditory bone (16). In our study, we investigated the HL13 proband was characterized by structural abnormalities of inner ear, X-linked recessive inheritance, and hemizygous deletion in *POU3F4*.

Our research resulted in a limited number of sporadic family samples, and the genetic results of some families are lack of validation by other affected family members, which needs a further evaluation from other relevant studies, especially those novel mutations. Our results also further verified the pathogenicity of the reported mutation sites in the hearing loss population.

## Conclusion

The results of the limited samples of this study show that hearing loss is a highly genetic heterogeneous disease. In hearing loss patients negative of *GJB2, SLC26A4*, and mitochondrial 12S rRNA, Usher and Waardenburg syndrome-related genes account for a major proportion in Chinese Han families, followed by *STRC* causing mild to moderate hearing loss.

## Data availability statement

The datasets presented in this study can be found in online repositories. The names of the repository/repositories and accession number(s) can be found at: https://www.ncbi.nlm.nih.gov/, PRJNA876030.

## Ethics statement

The studies involving human participants were reviewed and approved by the Ethics Committee of Xinhua Hospital affiliated to Shanghai Jiaotong University School of Medicine. Written informed consent to participate in this study was provided by the participants' legal guardian/next of kin.

## Author contributions

LS and ZL wrote the paper. XW, JS, YL, and YH collected and analyzed the data. JY and LS designed the research. All authors contributed to the article and approved the submitted version.

## Funding

This research was supported by grants from the National Natural Science Foundation of China, Grant/Award Numbers: 81873698.

## Conflict of interest

The authors declare that the research was conducted in the absence of any commercial or financial relationships that could be construed as a potential conflict of interest.

## Publisher's note

All claims expressed in this article are solely those of the authors and do not necessarily represent those of their affiliated organizations, or those of the publisher, the editors and the reviewers. Any product that may be evaluated in this article, or claim that may be made by its manufacturer, is not guaranteed or endorsed by the publisher.
