# Supplementary material for: Molecular etiology study of hearing loss in 13 Chinese Han families
Source: Front Neurol. 2022 Nov 23;13:1048218. doi: 10.3389/fneur.2022.1048218 (PMC9728030; doi:10.3389/fneur.2022.1048218)
Supplement: Supplementary file 1 [file Data_Sheet_1.docx]

**Supplementary Table S1.** 415 deafness genes targeted for the next-generation sequencing.


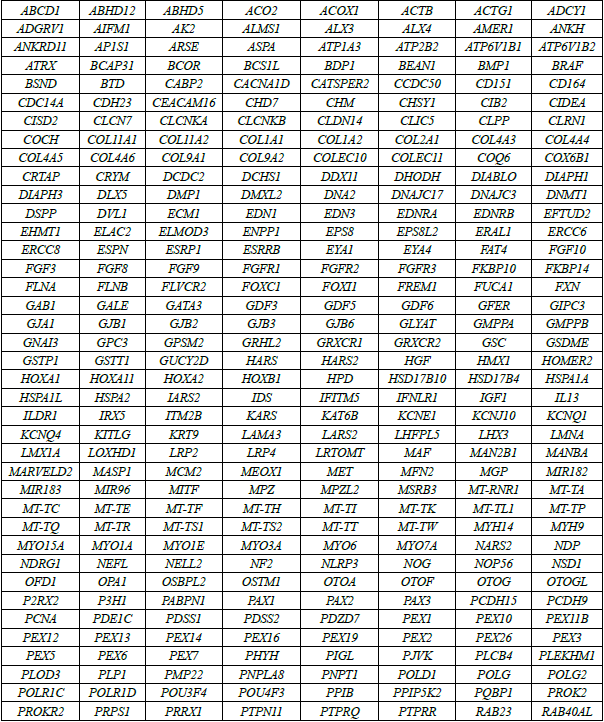

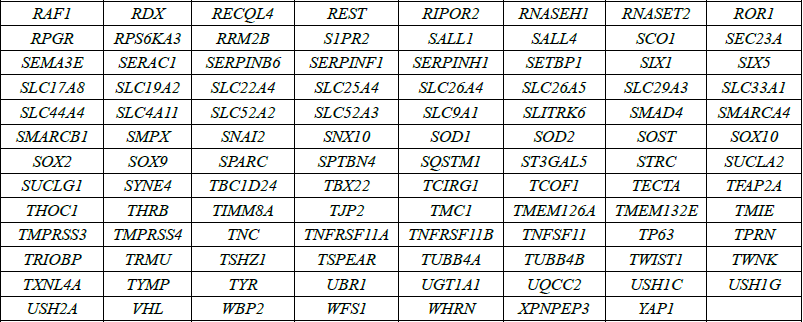


**Supplementary Table S2 Excluded of gene mutation sites**

| Family number | Gene | Nucleotide change (transcript version) | Amino acid change | Variant form | Mode of inheritance | Variation classification |
| --- | --- | --- | --- | --- | --- | --- |
| HL-1 | DIAPH1 | c.2200G>A (NM_005219) | p.G734R | Het | AD/AR | unknown |
|  | OSBPL2 | c.1376C>A (NM_144498) | p.T459N | Het | AD | unknown |
|  | MYO3A | c.3584T>C (NM_017433) | p.V1195A | Het | AR | unknown |
|  | ADGRV1 | c.6559A>G (NM_032119) | p.I2187V | Het | AR | unknown |
|  | CLCN7 | c.982-2A>G (NM_001287) |  | Het | AD/AR | Likely pathogenic |
|  | COL1A2 | c.2569C>A (NM_000089) | p.P857T | Het | AD/AR | unknown |
|  | PEX5 | c.1384C>T (NM_001131025) | p.L462F | Het | AR | unknown |
| HL-3 | USH2A | c.4637C>T (NM_206933.2) | p.Ala1546Val | Het | AR | unknown |
|  | PCDH15 | c.5254_5280del (NM_033056.3) | p.Pro1752_Pro1760del | Het | AR/ DR | unknown |
|  | OTOG | c.56G>A (NM_001277269.1) | p.Gly19Asp | Het | AR | unknown |
|  | KARS | c.1212_1220del (NM_001130089.1) | p.Asp404_Pro406del | Het | AR | unknown |
|  | SALL4 | c.427A>G (NM_020436.4) | p.Met143Val | Het | AD | unknown |
|  | TSPEAR | c.607C>T (NM_144991.2) | p.Arg203Trp | Het | AR | unknown |
|  | WFS1 | c.1406C>T (NM_006005.3) | p.Ser469Leu | Het | AD/ AR | unknown |
| HL-4 | PDZD7 | c.1267G>A (NM_001195263.1) | p.Ala423Thr | Het | AR | unknown |
|  | TYR | c.929dup (NM_000372.4) | p.Arg311Lysfs*7 | Het | AR | unknown |
|  | GAA | c.546+5G>T (NM_000152.4) |  | Het | AR | unknown |
|  | CLDN14 | c.295G>A (NM_144492.2) | p.Val99Ile | Het | AR | unknown |
|  | ADGRV1 | c.6886C>T (NM_032119.3) | p.Pro2296Ser | Het | AR | unknown |
|  | COL9A1 | c.845C>A (NM_001851.4) | p.Pro282His | Het | AR | unknown |
|  | EYA1 | c.679G>C (NM_000503.5) | p.Ala227Pro | Het | AD | unknown |
| HL-5 | LAMA3 | c.2374G>A (NM_198129) | p.V792I | Het | AR | unknown |
|  | OTOGL | c.4681A>G (NM_173591) | p.I1561V | Het | AR | unknown |
|  | TRIOBP | c.965C>T (NM_001039141) | p.A322V | Het | AR | unknown |
|  | USH2A | c.6998T>C (NM_206933) | p.V2333A | Het | AR | unknown |
| HL-7 | CABP2 | c.32G>A (NM_016366.2) | p.Arg11Gln | Het | AR | unknown |
|  | TECTA | c.235G>C (NM_005422.2) | p.Val79Leu | Het | AD/AR | unknown |
|  | MYO15A | c.3458G>A (NM_016239.3) | p.Arg1153His | Het | AR | unknown |
|  | PNPT1 | c.1562A>C (NM_033109.4) | p.Lys521Thr | Het | AR | unknown |
|  | LRP2 | c.2987G>A (NM_004525.2) | p.Arg996Gln | Het | AR | unknown |
|  | WHRN | c.1649C>G (NM_015404.3) | p.Thr550Ser | Het | AR | unknown |
| HL-9 | FGFR3 | c.616-3C>T (NM_000142) |  | Het | AD | unknown |
|  | ADGRV1 | c.18571G>C (NM_032119) | p.A6191P | Het | AD/AR | unknown |
|  | TCIRG1 | c.2464C>T (NM_006019) | p.P822S | Het | AR | unknown |
|  | MANBA | c.530A>G (NM_005908) | p.H177R | Het | AR | unknown |
|  | SLITRK6 | c.613G>C (NM_032229) | p.E205Q | Het | AR | unknown |
|  | MAN2B1 | c.1796G>T (NM_000528) | p.R599I | Het | AR | unknown |
|  | LHX3 | c.979G>A (NM_014564) | p.A327T | Het | AR | unknown |
|  | COL4A4 | c.4421C>T (NM_000092) | p.T1474M | Het | AD/AR | unknown |
| HL-10 | ELMOD3 | c.924C>G (NM_001135022) | p.D308E | Het | AR | unknown |
|  | GJB2 | c.109G>A (NM_004004) | p.V37I | Het | AR | unknown |
| HL-11 | TRIOBP | c.3652A>G (NM_001039141) | p.I1218V | Het | AR | unknown |
|  | USH2A | c.14570G>T (NM_206933) | p.G4857V | Het | AR | unknown |
| HL-12 | MEGF8 | c.1031A>G (NM_001410) | p.D344G | Het | AR | unknown |
|  | MEGF8 | c.4542G>C (NM_001410) | p.M1514I | Het | AR | unknown |
|  | WAC | c.1034T>C (NM_016628) | p.V345A | Het | AD | unknown |
|  | RIPOR2 | c.43T>C (NM_001286446) | p.W15R | Het | AR | unknown |
|  | ADCY1 | c.1789C>T (NM_021116) | p.R597W | Het | AR | unknown |
|  | CLIC5 | c.1217G>T (NM_001114086) | p.R406L | Het | AR | unknown |
|  | SUCLA2 | c.646A>G (NM_003850) | p.K216E | Het | AR | unknown |
| HL-13 | OTOG | c.169G>T (NM_001277269) | p.A57S | Het | AR | unknown |
|  | TMIE | c.419A>G (NM_147196) | p.K140R | Het | AR | unknown |
